# Pathological Findings of Postmortem Biopsies From Lung, Heart, and Liver of 7 Deceased COVID-19 Patients

**DOI:** 10.1177/1066896920935195

**Published:** 2020-06-19

**Authors:** Mohammad Taghi Beigmohammadi, Behnaz Jahanbin, Masoomeh Safaei, Laya Amoozadeh, Meysam Khoshavi, Vahid Mehrtash, Bita Jafarzadeh, Alireza Abdollahi

**Affiliations:** 1 48439 Tehran University of Medical Sciences, Tehran, Iran

**Keywords:** lung, postmortem examination, COVID-19, pathology, severe acute respiratory syndrome coronavirus 2

## Abstract

*Background*. A novel coronavirus named severe acute respiratory syndrome coronavirus 2 (SARS-CoV-2) has been affecting almost all nations around the world. Most infected patients who have been admitted to intensive care units show SARS signs. In this study, we aimed to achieve a better understanding of pathological alterations that take place during the novel coronavirus infection in most presumed affected organs. *Methods*. We performed postmortem core needle biopsies from lung, heart, and liver on 7 deceased patients who had died of coronavirus disease 2019. Prepared tissue sections were observed by 2 expert pathologists. *Results*. Diffuse alveolar damage was the main pathologic finding in the lung tissue samples. Patients with hospitalization durations of more than 10 days showed evidence of organization. Multinucleated cells in alveolar spaces and alveolar walls, atypical enlarged cells, accumulation of macrophages in alveolar spaces, and congestion of vascular channels were the other histopathologic alteration of the lung. None of our heart biopsy samples met the criteria for myocarditis. Liver biopsies showed congestion, micro- and macro-vesicular changes, and minimal to mild portal inflammation, in the majority of cases. *Conclusions*. Similar to the previous coronavirus infection in 2003, the main pathologic finding in the lung was diffuse alveolar damage with a pattern of organization in prolonged cases. The SARS-CoV-2 infection does not cause myocarditis, and the ischemia of myocardium is the most probable justification of the observed pathologic changes in the heart. Liver tissue sections mostly showed nonspecific findings; however, ischemia of the liver can be identified in some cases.

## Introduction

In the closing month of 2019, multiple cases of pneumonia of unknown etiology were reported in Wuhan city of Hubei province of China.^1^ A novel betacoronavirus called severe acute respiratory syndrome coronavirus 2 (SARS-CoV-2)—formerly named 2019-nCoV—was been identified as the causative agent, and the illness was named coronavirus disease (COVID-19) by the World Health Organization.^2,3^ The outbreak was eventually declared a pandemic in just about 3 months after emerging.^4^ Coronaviruses have induced respiratory tract and gastrointestinal infections in animals and humans.^5^ Despite the existence of 2 previous instances of coronavirus diseases, severe acute respiratory syndrome coronavirus (SARS-CoV) and the Middle East respiratory syndrome coronavirus (MERS-CoV), which had been identified as the causes of fatal diseases in humans, other coronaviruses have just induced mild flu-like symptoms in healthy individuals.^6^ The COVID-19 has given the impression of being a fatal coronavirus infection. It has been demonstrated that about 12% of patients presenting with severe symptoms require hospitalization and the case fatality ratio is about 2.3%.^7,8^ Clinical and laboratorial data on COVID-19 have identified lung, heart, and liver as the 3 organs involved by the novel coronavirus.^9^ Only a limited amount of data is available about pathological changes in patients infected by the SARS-CoV-2. Knowing about the underlying pathological alterations in the abovementioned organs can lead us to a better understanding of the pathophysiology of the disease and could be of importance in clinical management.

## Patients and Methods

Seven deceased patients in Imam Khomeini Hospital Complex of Tehran, Iran, with a diagnosis of SARS-CoV-2 infection were included in the study. The diagnoses were confirmed by the positive history of symptoms, chest computed tomography scan findings, as well as real-time polymerase chain reaction (RT-PCR) performed on secretions collected using nasopharyngeal and oropharyngeal swabs according to the World Health Organization interim guidance. RT-PCR tests were run on CFX96 Touch Real-Time PCR Detection Systems (Bio-Rad) by applying the Novel Coronavirus COVID-19 (2019 nCoV) Real Time Multiplex RT-PCR Kit (DaAn Gene Co, Ltd) and Novel Wuhan CoV E-gene kit (TIB Molbiol) by following the manufacturer’s instructions. Relevant clinical data and radiological reports were retrieved from patients’ files. After receiving ethical approval from Iran National Committee for Ethics in Biomedical Research with IR.TUMS.VCR.REC.1399.097 reference number and obtaining written informed consent from the legal guardians of the patients, multiple ultrasound-guided postmortem 14-gauge core-needle biopsies were taken from the lung, heart, and liver of the subjects. All tissue samples were taken within an hour after death and the procedures were performed in negative-pressure isolation rooms with at least 12 air changes per hour using appropriate personal protective equipment. Extracted tissue samples were immediately immersed in 10% neutral-buffered formalin and were sent to surgical pathology laboratory under standard laboratory biosecurity measures. After 24 hours of fixation, tissue processing was performed by using a vacuum automated tissue processor. Although coronavirus is expected to become noninfectious after exposure to high temperatures and ethanol during processing, yet samples were handled after wearing proper personal protective equipment.^10^ Lung, liver, and heart tissues were sectioned at 4 µm from paraffin-embedded tissue samples and stained using routine hematoxylin and eosin (H&E) stain. Immunohistochemical (IHC) staining for cytokeratin AE1/AE3, TTF1, leukocyte common antigen (LCA), CD20, CD3, and CD68 was analyzed in all lung tissue sections. IHC staining for LCA, CD68, and CD3 was performed on heart biopsies with more than 14 lymphocytes/macrophages per mm^2^ in H&E slides. All IHC antibodies were provided by Master Diagnóstica and were used according to the manufacturer’s protocols. Both suitable negative and positive controls were recruited to ensure that the obtained IHC staining results are accurate and valid. Furthermore, sections from liver tissue were stained using Masson’s trichrome and reticulin staining protocols. All staining procedures were conducted by well-trained technicians. Mounted slides were studied by 2 expert pathologists in the respective fields of thoracic and gastrointestinal pathology.

## Results

### Patient’s Characteristics

Seven deceased patients with confirmed diagnoses of SARS-CoV-2 infection were enrolled in our study. Our cases included 5 male and 2 female patients. The mean age of patients was 67.85 years (ranged from 46 to 84 years). The mean duration of hospitalization was 10 days (ranged from 3 to 19 days). All patients had been intubated with a mean duration of 5.4 days (ranged from 2 to 10 days). As shown in Table 1, fever and dyspnea were the main chief complaints at the time of admission. Four cases had suffered from high blood pressure and had taken oral medications. One of the patients was immunocompromised due to a long-term case of rheumatoid arthritis. This patient had been undergoing oral corticosteroid and Methotrexate therapy. Two of our cases had no previous underlying diseases. Computed tomography scan reports showed bilateral peripheral ground-glass opacities predominantly distributed along with basal segments in all the 7 cases.

**Table 1.** Clinical Features of Deceased COVID-19 Patients.

Patient	Age/sex (years)	Clinical presentation at admission	Past medical history	Drug history	Medications used for COVID-19	Duration of hospital stay (days)	Duration of intubation (days)
1	Male/58	Fever, dyspnea, nausea, and vomiting	HTN	Losartan, aspirin	HCQ, atazanavir	7	4
2	Female/84	Fever, dyspnea, myalgia	HTN	Amlodipine, aspirin, citalopram	HCQ, LPV/r^a^, oseltamivir	3	3
3	Female/72	Fever, headache, nausea, and vomiting	Rheumatoid arthritis	Sulfasalazine, prednisolone, MTX	HCQ, levofloxacin	15	6
4	Male/72	Fever, dyspnea, diarrhea	HTN, DM	Insulin^b^	HCQ, oseltamivir, azatanavir, levofloxacin	4	2
5	Male/68	Fever, dyspnea	HTN, valvular heart disease	Losartan, propranolol	HCQ, oseltamivir	19^c^	8
6	Male/46	Fever, dyspnea, myalgia, and sore throat	PUD	Chlordiazepoxide/clidinium	HCQ, remdesivir, naproxen, cefepime	16	10
7	Male/75	Fever, dyspnea, anorexia	None	None	HCQ, oseltamivir	6	5

Abbreviations: HTN, hypertension; HCQ, hydroxycholorquine; LPV/r, lopinavir/ritonavir (Kaletra); MTX, methotrexate; DM, diabetes mellitus; PUD, peptic ulcer disease.

a Tamiflu.

b NovoRapid and Lantus.

c This patient was admitted to hospital due to endocarditis and had undergone heart valve replacement surgery. Respiratory symptoms revealed about 11 days before expiration.

### Pathologic Findings of Lung Tissue

**Figure 1.**
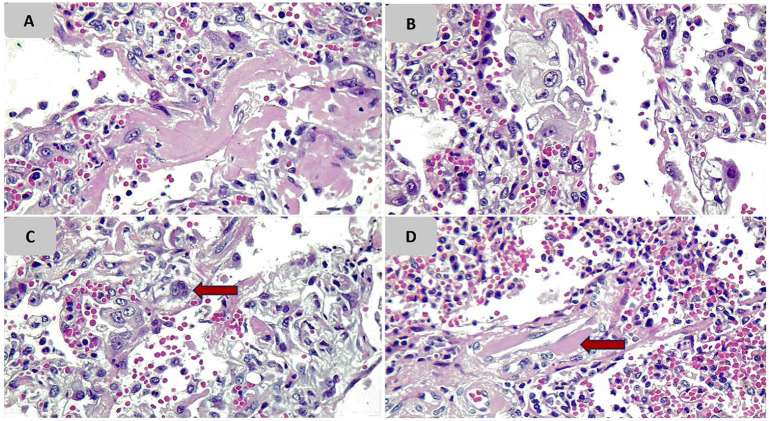
Pathologic findings of lung tissue of case 1. (A) Hyaline membrane formation; (B) intra-alveolar atypical enlarged cells; (C) multinucleated cells; (D) deposition of fibrinoid material in vessel walls.

**Figure 2.**
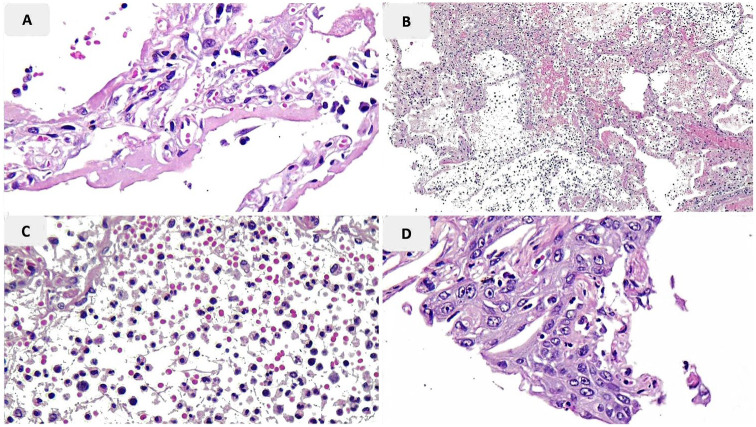
Pathologic findings of lung tissue of case 7. (A) Hyaline membrane formation; (B, C) necrosis and acute inflammatory reaction; (D) squamous metaplasia of bronchiole.

A summary of lung biopsy findings is demonstrated in Table 2. Histologic evidence of the diffuse alveolar damage including hyaline membrane formation, alveolar wall edema, and fibrin exudate was present in 5 patients (cases 1, 2, 4, 5, and 7; Figures 1 and 2).

**Table 2.** Pulmonary Pathologic Findings of Deceased COVID-19 Patients.

	Patient						
	1	2	3	4	5	6	7
Hyaline membrane formation	Present	Present	NI	Present	Present	NI	Present
Edema	Present	Present	Present	Present	Present	Present	Present
Fibrin exudation	NI	Present	Present	NI	Present	Present	Present
Multinucleation	Present	Present	Present	NI	Present	Present	NI
Inflammation intensity	Severe in 1 out of 4 cores but mild in other cores	Mild	Severe	Mild and focal	Moderate	Severe	Severe
Inflammation site	Alveolar walls	Alveolar walls	Alveolar spaces	Alveolar wall	Alveolar walls and alveolar spaces	Alveolar space and alveolar walls	Alveolar spaces and alveolar walls
Predominant type of inflammatory cells	Lymphocytes and plasma cells with few PMNs	Lymphocytes with occasional PMNs	PMNs and few lymphocytes	Lymphocyte and rare PMNs	Lymphocytes and few PMNs	PMNs and lypmhocytes	PMNs and lymphocytes
Pneumocyte type II hyperplasia	Present	NI	Present	NI	Present	Present	Present
Atypical enlarged cells	Present	Present	Present	Present	Present	Present	Present
Acute pneumonia pattern	NI	NI	Present	NI	NI	Present	Present with necrosis
Organization	NI	NI	Present	NI	Present	Present	NI
Accumulation of macrophages in alveolar spaces	Present	Present	Present	NI	Present (hemosiderin-laden macrophages are also seen)	Present	NI
Fresh hemorrhage	Present	NI	NI	NI	NI	Present	NI
Vessels	Fibrinoid material deposition in vessel walls	Fibrinoid material deposition in vessel walls	No obvious pathological finding	No obvious pathological finding	No obvious pathological finding	No obvious pathological finding	Fibrinoid material deposition in vessel walls
Squamous metaplasia	No obvious bronchioles	NI	NI	No obvious bronchioles	Present, associated with bronchiolitis	No obvious bronchioles	NI

Abbreviation: NI, not identified; PMN, polymorphonuclear neutrophils.

**Figure 3.**
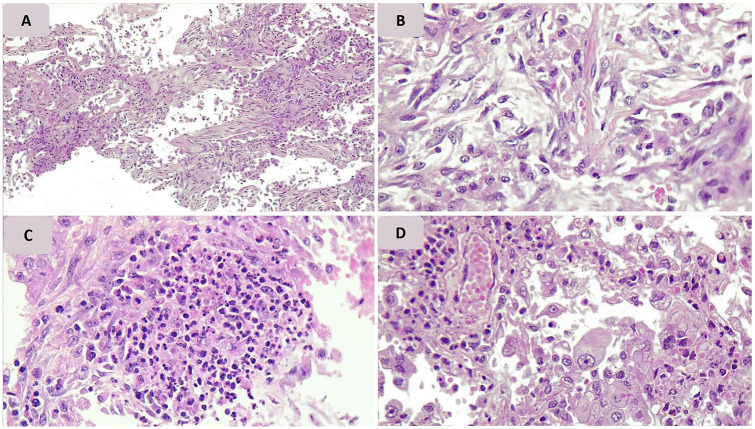
Pathologic findings of lung tissue of case 3. (A, B) Proliferation of fibroblasts (organizing diffuse alveolar damage); (C) acute pneumonia; (D) enlarged atypical cell.

Type II pneumocyte hyperplasia and interstitial fibroblastic proliferation, which can be histologic signs of the organizing phase of pneumonia or the organizing phase of diffuse alveolar damage (DAD), were noted in 3 cases with prolonged durations of hospitalization of more than 10 days (cases 3, 5, and 6; Figure 3). In 2 of them, histologic features of acute pneumonia were observed without evident hyaline membrane formation. Only in one of them (case 5) concurrent evidence of focal hyaline membrane formation was present.

Atypical enlarged cells with large nuclei, prominent eosinophilic nucleoli, and remarkable vacuolated and acidophilic cytoplasm were seen in all patients. These cells were stained with CKAE1/AE3 and TTF1 markers in IHC staining that pointed to their pneumocyte origin.

In one case (case 1), a severe inflammatory reaction with a predominance of lymphocytes was observed in the alveolar wall and interstitium in one of the biopsy cores, which was associated with fresh hemorrhage. However, in the remaining 3 biopsy cores of case 1, evidence of DAD was obvious.

In 3 cases (cases 3, 6, and 7) marked intra-alveolar accumulation of neutrophil-rich inflammatory cells was observed, which is compatible with acute pneumonia.

In 2 cases (cases 2 and 4) inflammatory infiltration was scant and predominantly limited to alveolar walls, and the hyaline membrane was present in both cases.

Accumulation of macrophages in alveolar spaces was observed in all cases and in some of them was admixed with desquamated type II pneumocytes.

Due to the limited biopsy cores of lung tissue, definite assessment of small airways was not possible; however, squamous metaplasia in 2 cases (cases 5 and 7) in combination with mild infiltration of mixed inflammatory cells was noted.

**Figure 4.**
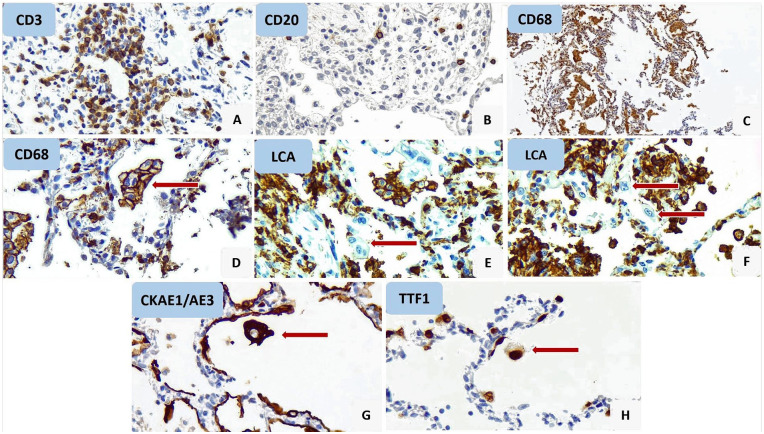
Lung tissue samples immunohistochemistry study results. (A) CD3 stained major proportion of lymphocytes; (B) CD20 stained few lymphocytes; (C) CD68, a histiocytic marker, stained major population of intra-alveolar cells; (D) CD68 stained some multinucleated cells; (E) some multinucleated cells did not stain with LCA; (F) large atypical cells (arrow) did not stain with LCA; (G, H) large atypical cells stained with CKAE1/AE3 and TTF1.

Predominant inflammatory cells were macrophages and T-lymphocytes and this finding was confirmed by IHC staining for CD68 and CD3 (Figure 4).

### Pathologic Findings of Heart Tissue

As depicted in Table 3, 5 out of 7 heart necropsies contained cardiac muscle tissue. Heart tissue was not observed in necropsy samples of cases 2 and 4.

**Table 3.** Heart Pathologic Findings of Deceased COVID-19 Patients.

	Patient						
	1	2	3	4	5	6	7
Interstitial inflammation	Present	No heart tissue	Present	No heart tissue	Present	Not identified	present
Intensity of inflammation	Focal (<4/mm^2^)	—	Mild to moderate (>20/mm^2^)	—	Severe (>34/mm^2^)	N/A	Mild to moderate (20/mm^2^)
Myocyte necrosis	Not seen	—	Not seen	—	present	N/A	
Vasculitis	Not seen	—	Not seen	—	Not seen	N/A	
LCA positive cells	N/A	—	>23/mm^2^	—	>34/mm^2^	N/A	>24/mm^2^
CD3 positive cells	N/A	—	Negative	—	<10/mm^2^	N/A	<4/mm^2^
CD68 positive cells	N/A	—	>23/mm^2^	—	>24/mm^2^	N/A	>20/mm^2^

Abbreviations: N/A, not applicable; LCA, leukocyte common antigen.

IHC staining was performed using LCA, CD3, and CD68 to highlight leukocytes, T-lymphocytes, and macrophages, respectively, on cases 3, 5, and 7, in which H&E-stained tissue sections demonstrated histologic evidence that could be interpreted as myocarditis.

Case 1 revealed few scattered lymphocytes and mastocytes without evidence of myocyte necrosis or degeneration, and as such, did not meet the criteria for myocarditis.

In case 3, all inflammatory cells demonstrated positive immunoreactivity for CD68; however, none of them was stained with CD3. So, the diagnosis of myocarditis was excluded and although evidence of myocyte necrosis was not present, the ischemic process of cardiac muscle was highly suggested.

**Figure 5.**
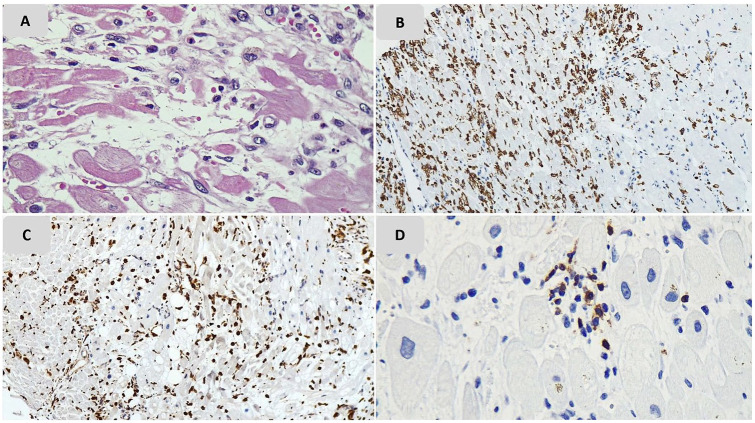
Pathologic findings of heart tissue biopsy of case 5.(A) Interstitial inflammatory infiltrate with ischemic necrosis; (B) immunohistochemical (IHC) staining for LCA; (C) IHC staining for CD68; (D) IHC staining for CD3.

In case 5, severe interstitial infiltration of LCA-positive inflammatory cells was obvious with a predominance of CD68 positive macrophages and focal aggregation of CD3 positive T-cells. Histologic evidence of myocyte necrosis including hypereosinophilia and enucleation were seen. This patient had undergone a heart valve replacement procedure due to endocarditis about 1 month before his death. Regarding medical history and histopathologic findings, the ischemic necrosis of myocardium should be considered (Figure 5).

In case 7, the majority of inflammatory cells showed immunoreactivity for CD68 and rare cells were positive for CD3. So, the diagnosis of myocarditis was not established and although evidence of myocyte necrosis was not evident, the ischemic process of cardiac muscle was highly suggested.

### Pathologic Findings of Liver Tissue

**Figure 6.**
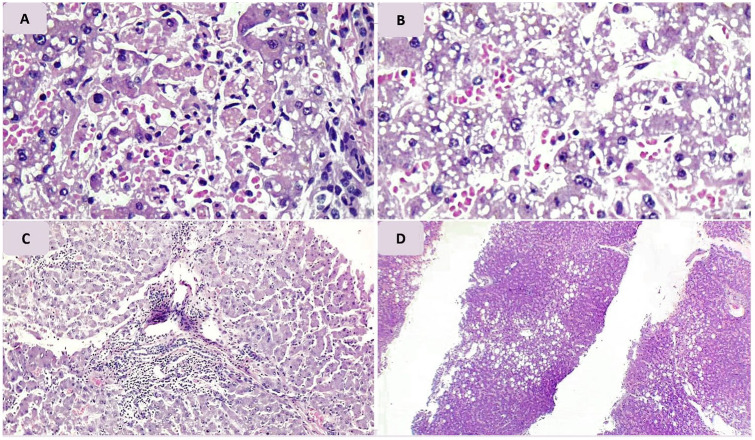
Pathologic findings of liver tissue samples. (A) Focal confluent necrosis (case 7); (B) moderate microvesicular changes (case 5); (C) mild portal tract mixed inflammation with focal interface hepatitis (case 1); (D) mild steatosis (predominantly macrovesicular) involves about 30% of lobular area admixed with mild microvesicular change (case 2).

As shown in Table 4, all of our cases had congestion and sinusoidal dilation sinuses in liver sections ranged from mild to severe forms. Minimal to mild micro- and macrovesicular steatosis was evident in almost all liver sections. Four cases had mild ballooning degeneration of hepatocytes. Focal and scattered bile plugs were observed in 2 patients. In case 7, focal confluent necrosis was observed, and in case 4 focal hepatocyte drop out was evident. Masson’s trichrome special stain showed rather normal findings. Reticulin stain revealed areas of focal regeneration in 5 patients. No evidence of the viral cytopathic effect was observed in the liver tissue sections (Figure 6).

**Table 4.** Liver Pathologic Findings of Deceased COVID-19 Patients.

Patient	Number of portal tracts in biopsy	Portal inflammation	Interface hepatitis	Confluent necrosis	Focal lytic necrosis, apoptosis, and focal inflammation	Trichrome stain	Reticulin stain	Congestion	Other findings
1	15	Mild	Mild	Absent	One focus or less per 10× objective	Unremarkable	Focal regeneration	Mild	Mild macrovesicular steatosis involves about 5% of lobular area, and mild microvesicular change.
2	13	Minimal	Absent	Absent	One focus or less per 10× objective	Unremarkable	Unremarkable	Severe	Mild macrovesicular steatosis involves about 30% of lobular area, mild microvesicular, and mild ballooning degeneration.
3	4	Mild	Mild	Absent	One focus or less per 10× objective	Mild fibrosis	Focal regeneration	Moderate	Minimal macrovesicular steatosis involves less than 5% of lobular area and scattered bile plugs.
4	15	Mild	Absent	Absent (but Focal hepatocyte drop out is seen)	One focus or less per 10× objective	Unremarkable	Unremarkable	Mild	Minimal macrovesicular steatosis involves about 5% of lobular area and mild microvesicular changes.
5	13	Mild	Mild	Absent	One focus or less per 10× objective	Unremarkable	Unremarkable	Moderate	Mild ballooning degeneration and focal bile plug
6	27	Minimal	Absent	Absent	One focus or less per 10× objective	Unremarkable	Focal regeneration	Moderate to severe	Mild macrovesicular steatosis involves about 30% of lobular area, mild microvesicular changes, and mild ballooning degeneration.
7	15	Mild	Mild	Present (focal confluent necrosis in acinar zone 3 and 2 is seen)	One focus or less per 10× objective	Unremarkable	Focal regeneration	Moderate	Minimal macrovesicular steatosis involves about 5% of lobular area, moderate microvesicular changes, and mild ballooning degeneration.

## Discussion

Most of the pathologic findings in postmortem lung biopsies of 7 deceased SARS-CoV-2 infected patients in our study demonstrated the acute phase of DAD. The acute phase of DAD is associated with hyaline membrane formation, alveolar wall edema, and fibrinoid exudate.^11^ Proliferative and fibrotic phases, more advanced stages of DAD, have also been described.^12^

This finding is in line with the pathologic findings of a previous pandemic of SARS due to coronavirus in 2003 and also Middle East respiratory syndrome coronavirus infection of 2014. In previous studies on patients with SARS and MERS, DAD was the main pathological observation.^13,14^

Tian et al^15^ presented the first pathologic description of lungs in 2 individuals who were diagnosed with COVID-19 following surgery due to lung cancer. Their findings demonstrated the early phase of the disease in asymptomatic patients, which matched the early phase of DAD, including proteinaceous exudate, edema, and pneumocystic hyperplasia with mononuclear inflammatory cells infiltration.

Also, they presented a pathological report of lungs in 4 deceased COVID-19 patients, referring to the abovementioned findings, plus hyaline membrane formation, establishing DAD as the pathologic basis of lung involvement in COVID-19 patients.^16^

Similarly, DAD was the main finding in a case reported by Xu et al^17^ of a patient who had died of COVID-19. Hyaline membrane formation, desquamated pneumocytes, presence of mononuclear dominant leukocytes, and pulmonary edema were mentioned in their study.

In our study, histologic evidence of organization was seen in 3 cases with hospitalization durations of more than 10 days. Only in one of them, hyaline membrane formation was observed, and in 2 others, histopathologic findings of acute pneumonia without hyaline membrane formation were present. The insufficiency of lung tissue samples may have restricted the finding of the hyaline membrane in the latter cases. So, we can conclude that a correlation was observed between the duration of disease and histologic findings of organization (fibroblastic proliferation and occasional intra-alveolar papillary projections).^18^ A study by Franks et al^14^ about the pathological alteration in patients infected with SARS-CoV during the 2003 pandemic confirmed DAD as the primary pathology of lung. They stated that patients with different periods of infection showed distinct histopathological findings. Acute and organizing patterns of DAD were observed in patients with less and more than 10 days of clinical course, respectively, as we have shown in the present study.

In our study, atypical enlarged cells that are reactive pneumocytes were seen in all of our lung tissue samples. These cells showed positive results on CK AE1/AE3 and TTF1 immunostaining. Some of them closely resembled a viral cytopathic effect. The presence of atypical cells was described in another case report on a patient with COVID-19.^17^

Multinucleated cells were seen in alveolar spaces and, to a lesser extent, in lung interstitium of 5 biopsy samples from our cases. Multinucleated cells were also reported in patients infected with previous SARS coronavirus. Nicholls et al^19^ suggested that these cells arise from macrophages and epithelial cells. In our study, these cells showed positive results in CD68 and CKAE1/AE3 IHC staining which indicated the same results about the origin of the cells as the previous studies. The presence of multinucleated cells has been well established as a characteristic pathologic evidence of coronavirus-induced pneumonia and has been also observed in patients diagnosed with COVID-19.^17,20^

Squamous metaplasia was seen in 2 of our cases, one of which demonstrated simultaneous organizing pattern and the other showed evidence of acute necrotizing pneumonia. Previous pathologic findings in cases with fatal human influenza A (H1N1) have alluded to intense epithelial squamous metaplasia in large airways. Concordantly, these findings were seen in the early stages of infection with SARS coronavirus in 2003.^19,21^

Major inflammatory cells in our biopsy samples taken from lung tissue of COVID-19 patients were macrophages and T-lymphocyte.

In 3 of our cases, intra-alveolar accumulation of polymorphonuclear leukocytes associated with necrosis was observed. This finding can be interpreted as acute pneumonia resulting from superimposed bacterial infection.^22^ Assessment of patients for concurrent superimposed bacterial infection, especially in those with longer hospital stay durations, seems to be necessary to devise appropriate treatment plans.

According to the qualitative Dallas criteria and Consensus paper of the European Society of Cardiology Working Group on Myocardial and Pericardial disease, myocarditis is defined as an inflammation of the myocardium associated with the presence of non-ischemic necrosis or degeneration of myocytes. Endomyocardial biopsy is the established gold standard procedure for the diagnosis of myocarditis. Supplementary tests, especially IHC staining, may contribute to an increase in sensitivity of diagnosis.

Presence of more than 14 leukocytes per mm^2^ in the myocardium including up to 4 monocytes per mm^2^, and detection of 7 or more CD3 positive T-lymphocytes are required for histologic diagnosis of myocarditis.^23,24^

In our cases, only 5 out of 7 necropsies contained cardiac muscle. In 3 cases (3/5) interstitial infiltration of mononuclear inflammatory cells were observed. Considering the criteria of myocarditis, none of our cases could be identified as myocarditis. Although more than 14 leukocytes per mm^2^ were present in our cases (confirmed by IHC staining for LCA), due to predominance of macrophages (CD68 positive) in all 3 cases, a scant number of CD3 positive T-lymphocytes, and also the presence of ischemic-type necrosis of myocytes in one of the cases, we concluded that ischemia of myocardium is the most probable diagnosis among our patients. Myocardial ischemia can be explained by the presence of hypoxia in the clinical setting of these patients due to severe lung involvement. Similarly, other studies about COVID-19 patients showed no obvious pathologic alteration in the patient’s heart, proposing that heart tissue is not directly invaded by SARS-CoV-2.^16,17^

Based on laboratory findings (serum liver enzyme and bilirubin levels), liver dysfunction among patients with COVID-19 has been reported in 14.8% to 53% of the cases in different studies.^25^ But, the prevalence of increased enzymes is higher in severe cases. In our study, all cases had an increased level of serum liver enzymes. The exact mechanism of liver damage during the novel coronavirus infection is not clear; however, drug toxicity and systemic inflammatory response have been proposed. In the current study, all of our liver biopsy samples taken from deceased COVID-19 patients had pathologic features of hepatic congestion ranged from mild to severe. Severe respiratory diseases resulting from SARS-Cov-2 infection could lead to right ventricular failure and the resultant passive hepatic venous congestion.^24,26^

A study on patients infected with coronavirus in 2003 showed frequent mitoses and the presence of high proliferation of hepatocytes; however, in our liver tissue samples from patients infected with novel coronavirus, we did not observe such instances.^27^

Focal confluent necrosis and hepatocyte drop out/necrosis in 2 of our patients could be related to the ischemic liver injury or virus-induced changes. Considering little inflammation in portal tracts and lobules in both of these cases as well as the pattern of liver necrosis in acinar zones 3 and 2, ischemic injury was highly suggested. Also, case 7 had myocyte necrosis in heart biopsy sample in favor of ischemia, which was similar to liver histologic findings; nevertheless, the possibility of drug-induced liver injury cannot be completely excluded in these patients. We did not have a heart biopsy sample of case 4 to compare with liver changes. Although sepsis in COVID-19 patients is considered as one of the causes of liver injury, liver biopsies in our patients did not show characteristic microscopic features of sepsis such as ductular cholestasis.

In general, no significant lobular and portal inflammation was identified in our cases. Likewise, observed histopathologic alterations in liver biopsy samples of our cases, including micro- and macrovesicular changes, mild portal inflammation, and focal interface hepatitis in the minority of portal tracts were non-specific findings.

Our findings are in line with observations mentioned in former studies. Liver biopsies in COVID-19 patients manifested microvesicular steatosis, portal, lobular, and sinusoidal inflammation, and multifocal necrosis which was interpreted as either a COVID viral-induced injury or a drug-induced hepatic insult.^17,28^

In conclusion, we demonstrated that in patients who were infected with the SARS-CoV-2 virus, DAD was the major histologic findings in the lung. The longer the duration of disease the higher the likelihood of identification of the organizing pattern. Atypical large cells were present in the lung of all of our cases, which may be reactive pneumocytes or, at least in some, may indicate the presence of a viral cytopathic effect. In our cases, evaluation of heart tissue samples did not confirm the diagnosis of myocarditis, and ischemia or presence of underlying heart diseases could be the cause of observed pathologic alterations. Liver biopsy samples demonstrated mostly a range of non-specific pathologic findings.
